# Knockdown of ribosomal protein S15A induces human glioblastoma cell apoptosis

**DOI:** 10.1186/s12957-016-0891-8

**Published:** 2016-04-29

**Authors:** Chen Zhang, Jiqiang Fu, Fei Xue, Bomi Ryu, Ting Zhang, Shuili Zhang, Jingyu Sun, Xinxin Xu, Zhaoli Shen, Longpo Zheng, Xianzhen Chen

**Affiliations:** Shanghai Tenth People’s Hospital, Tongji University School of Medicine, 301 Yanchang Road, Shanghai, 200072 China; Shanghai Municipal Hospital of Traditional Chinese Medicine, Shanghai University of Traditional Chinese Medicine, 274 Middle Zhijiang Road, Shanghai, 200071 China; College of Medical Technology, Zhejiang Chinese Medical University, Hangzhou, 310053 China; College of Pharmacy, Zhejiang Chinese Medical University, Hangzhou, 310053 China; Sports and Health Research Center, Tongji University Department of Physical Education, Shanghai, 200092 China

**Keywords:** Glioblastoma, RPS15A, Cell proliferation, Apoptosis

## Abstract

**Background:**

RPS15A is a ribosome protein that is highly conserved in many organisms from yeast to human. A number of studies implied its role in promoting cancer cell growth.

**Methods:**

Here, we firstly conducted RPS15A gene expression analysis in brain cancer using Oncomine database and found RPS15A was remarkably overexpressed in glioblastoma (GBM) compared with that in normal tissues. Then, the expression of RPS15A was specifically silenced in GBM cell line U251 using lentiviral-mediated RNA interference technique. We further investigated the effect of RPS15A knockdown in U251 cells using MTT assay, colony formation test, and flow cytometry analysis. We detected the protein level of Bcl-2 and poly (ADP-ribose) polymerase (PARP) as well as activation of caspase-3.

**Results:**

Our results showed that the knockdown of RPS15A could inhibit cancer cell growth and colony formation in vitro, as well as induced cell cycle arrest at G0/G1 phase and cell apoptosis. In addition, Western blot analysis indicated that the knockdown of RPS15A could significantly inhibit bcl-2 and activate caspase-3 and PARP.

**Conclusions:**

Our findings suggest RPS15A may play an important role in the progression of GBM and lentiviral-mediated silencing of RPS15A could be an effective tool in GBM treatment.

## Background

Glioma is the most common and most aggressive malignant brain tumor in the human central nervous system. According to histological progression, gliomas are classified into four grades, including low-grade astrocytomas (grade I–II), anaplastic astrocytomas (grade III), and glioblastoma (GBM, grade IV) [1, 2]. As a frequent form of malignant glioma [3], GBM is invariably lethal for patients at most 2 years following diagnosis [4]. Current standard treatment for patients with GBM is concurrent TMZ and radiotherapy followed by six maintenance cycles of adjuvant chemotherapy to improve progression-free survival and overall survival [5]. Despite the standard of care therapy, recurrence rates remain higher in patients with GBM. Therefore, it is urgent to identify new molecular targets to improve the prognosis against human GBM.

Ribosome is the fundamental translational machinery for protein synthesis from messenger RNAs. In eukaryotes, a mature ribosome consists of 60S and 40S subunits [6]. Ribosome biogenesis and protein translation are fine-tuned to match the cell growth rate, proliferation, and differentiation during development. However, this type of regulation is abnormal in cancer cells [7]. Several tumor suppressors could inhibit tumor cell growth by affecting ribosome biogenesis [8]. On the other hand, oncogenes often stimulate cell growth and proliferation by enhancing ribosome biogenesis in cancer cells [9]. RPS15A, ribosomal protein s15a, is one of the subunits of the 40S ribosomal protein [10, 11]. To data, there are several preliminary studies which show that down-regulation of RPS15A suppresses proliferation of lung cancer cells, osteosarcoma cells, and hepatic cancer cells in vitro [12–14]. Despite the ubiquitous presence and important functions of ribosomal complex, the role of ribosomal protein RPS15A in GBM cells remains not to be fully understood.

Herein, we aimed to investigate the effect of RPS15A knockdown in GBM progression. We firstly found the messenger RNA (mRNA) levels of RPS15A were significantly up-regulated in glioma compared that in normal tissues using the datasets from Oncomine database. Functional analysis showed knockdown of RPS15A obviously suppressed cell proliferation in U251 by inducing cell cycle arrest and apoptosis. More interestingly, we found RPS15A knockdown induced apoptosis through cell intrinsic mitochondria pathway. Our results suggested a prominent role of RPS15A in GBM cell proliferation and provide a new insight to a potential therapeutic target for GBM.

## Methods

### Cell lines and culture

The human GBM cell line U251 and the human embryonic kidney cell line 293T (HEK293T) were purchased from Cell Bank of Chinese Academy of Science (Shanghai). Cells were cultured in DMEM (Hyclone) supplemented with 10 % FBS (Biological Industries) at 37 °C in an incubator containing 5 % CO_2_.

### Construction of RPS15A short hairpin RNA containing lentivirus

For the RNA interfering experiment, two short hairpin RNA (shRNA) sequences were designed against the RPS15A gene (NM_001019): shRPS15A-1, 5′-GTGCAACTCAAAGACCTGGAACTCGAGTTCCAGGTCTTTGAGTTGCACTTTTT-3′ and shRPS15A-2, 5′-GCATGGTTACATT-GGCGAATTCTCGAGAATTCGCCAATGTAACCATGCTTTTT-3′). The control shRNA (shCon) sequence was 5′-GCGGAGGGTTTGAAAGAATATCTCGAGATATTCTTTCAAACCCTCCGCTTTTTT-3′. Each nucleotide sequence was synthesized and inserted into pFH-L shRNA expressing vector. For lentiviral production, recombinant plasmid described above and pHelper plasmids (pVSVG-1 and pCMVΔ8.92) were co-transfected into HEK293T cells using Lipofectamine 2000 (Invitrogen) according to the manufacturer’s instruction. After 72 h, virus supernatant was harvested and concentrated by ultracentrifugation (4000*g* at 4 °C) for 10 min.

### Cell infection

For cell infection, U251 cells were seeded at a density of 50,000 cells per well in six-well plates and transduced with constructed lentiviruses (shCon, shRPS15A-1, and shRPS15A-2) at a multiplicity of infection of 40. After 72-h infection, the infection efficiency was determined by observing the green fluorescence protein (GFP) expression under a fluorescence microscope.

### Quantitative PCR analysis

After 6-day infection, total RNA was isolated from cells by using the Trizol reagent (Invitrogen), according to the manufacturer’s protocol. Reverse transcribed was performed Bio-Rad Connect Real-Time PCR (polymerase chain reaction) platform (Bio-Rad Laboratories Inc, Hercules, CA, USA) using an SYBR Green Master Mix Kit. Data were analyzed using the 2^ΔΔCt^ method, and the levels of mRNA were normalized to β-actin. The PCR primers used were as follows: RPS15A (forward-CCTCCTTTTTCGGTTTCCTC; reverse-AGAGATGGAA-TGGTGGTTGG); β-actin (forward-GTGGACATCCGCAAAGAC; reverse-AAAGGGTGT-AACGCAACTA).

### Western blot analysis

Western blot analysis was done 5 days post infection. Proteins were extracted from cells using 2× SDS sample buffer (100 mM Tris-HCl (pH 6.8), 10 mM EDTAm 4 % SDS, 10 Glycine. A total of 30 ug proteins were separated in 12 % SDS-PAGE and transferred to PVDF membranes. The membranes were incubated with primary antibodies (rabbit anti-RPS15A, 1:1000, Ab175054, Abcam; rabbit anti-caspase-3, 1:500, #9661, Cell signaling; rabbit anti-poly (ADP-ribose) polymerase (PARP), 1:1000, #9542, Cell signaling; rabbit anti-bcl-2, 1:1000, #2876, Cell signaling; rabbit anti-GAPDH, 1:500000, #2876, Cell signaling) for 1 h at room temperature followed by incubation with secondary antibody goat anti-rabbit HRP (Santa Cruz) at room temperature for 1 h. The bands were visualized using an ECL kit (Beyotime). Protein bands were quantified using Gel-pro analyzer software (MediaCybernetics). GAPDH was used as the reference control.

### MTT assay

Cells were seeded in a 96-well plate with 2500 cells per well. Growth curve and MTT assay was carried out 96 h post transduction of virus. Briefly, MTT solution was added to each well, followed by 4 h of incubation at 37 °C. Then, cells were washed and dissolved in acidic isopropanol (10 % SDS, 5 % isopropanol, and 0.01 mol/L HCl) for 10 min. Cell density was measured at 595 nm using the microplate reader using enzyme-linked immunosorbent assay. The cell growth curves were drawn according to the OD values.

### Colony formation analysis

Cells were seeded at the density of 500 cells per well in a six-well plate. After 96 h infection with shRNA virus, followed by additional eight days of incubation, cells were washed with PBS twice, fixed with absolute methanol for 15 min. Then, fixed cells were stained with 1 % crystal violet for 20 min. After cleaning with PBS, colonies were counted under light microscope.

### Cell cycle and apoptosis analysis

Cells were seeded in a 6-cm dish at 100,000 cells per well. Four days after infection with lentivirus, the cells were fixed with pre-cooled 70 % ethanol at 4 °C overnight and incubated with 1 mg/ml RNase A (QIAGEN) for 30 min at 37 °C. Then, cells were added propidium iodide (50 ug/mL, ebioscience) at 4 °C for 30 min to stain DNA. The DNA content of cells was determined by a FACS Calibur II sorter and Cell Quest FACS system (BD Biosciences).

For cell apoptosis analysis, cells were harvested after 3-day infection with lentivirus and resuspended in 100 ul 1× binding buffer (ebioscience). Then, cells were stained with 2 uL Annexin V-APC (20 ug/ml; ebioscience) for 15 min on ice. Samples were diluted to 400 uL and added 1 uL 7-AAD (50 ug/ml; ebioscience) before detection on FACS Calibur II sorter and Cell Quest FACS system (BD Biosciences).

### Statistical analysis

All experiments were at least repeated in triplicate. All data were analyzed using GraphPad Prism software and expressed as the mean ± standard deviation (SD) of three independent experiments. Comparisons between groups were conducted by Student’s *t* test. The *p* value <0.05 was considered as statistically significant.

## Results

### RPS15A is overexpressed in GBM

In order to evaluate the differential expression of RPS15A in GBM, we used the Oncomine database (www.oncomine.org), a cancer microarray database of genome-wide expression analysis [15]. A quick search of RPS15A gene in GBM revealed that this gene was up-regulated in several types of GBM, including anaplastic oligoastrocytoma [16, 17], glioblastoma [16, 18], oligodendroglioma [19], and diffuse astrocytoma [20] vs. normal tissues (Table 1). The comparison showed an average increase of one- to sixfold in RPS15A expression in GBM compared to normal tissues. Therefore, we concluded that RPS15A might play an important role in GBM progression. Thus, we focused on this particular gene in following studies by using U251, a cell line derived from a malignant glioblastoma.

**Table 1:** RPS15A differential transcript expression in human glioblastoma extracted from multiple studies in the Oncomine database

Study	Comparison (specimen number in each group)	Fold change	*p* value
Bredel M et al.	Anaplastic oligoastrocytoma (6) vs. normal(4)	2.125	0.005
Bredel M et al.	Anaplastic oligodendroglioma (3) vs. normal (4)	2.337	0.004
Bredel M et al.	Glioblastoma (5) vs. normal (4)	1.916	6.64E−04
Bredel M et al.	Oligodendroglioma (27) vs. normal (4)	2.014	0.006
French PJ et al.	Anaplastic oligodendroglioma (23) vs. normal(6)	2.373	4.09E−05
Murat A et al.	Glioblastoma (80) vs. normal(4)	1.985	1.51E−08
Shai R et al.	Oligodendroglioma (3) vs. normal (7)	5.803	5.49E−06
Sun L et al.	Diffuse astrocytoma (7) vs. normal (23)	2.163	8.40E−07
TCGA	Brain glioblastoma (542) vs. normal (10)	1.408	7.04E−04

*TCGA* The Cancer Genome Atlas-Glioblastoma Multiforme Gene Expression Data (http://tcga-data.nci.nih.gov/tcga/)

### RPS15A gene knockdown in U251 cells

**Fig. 1:**
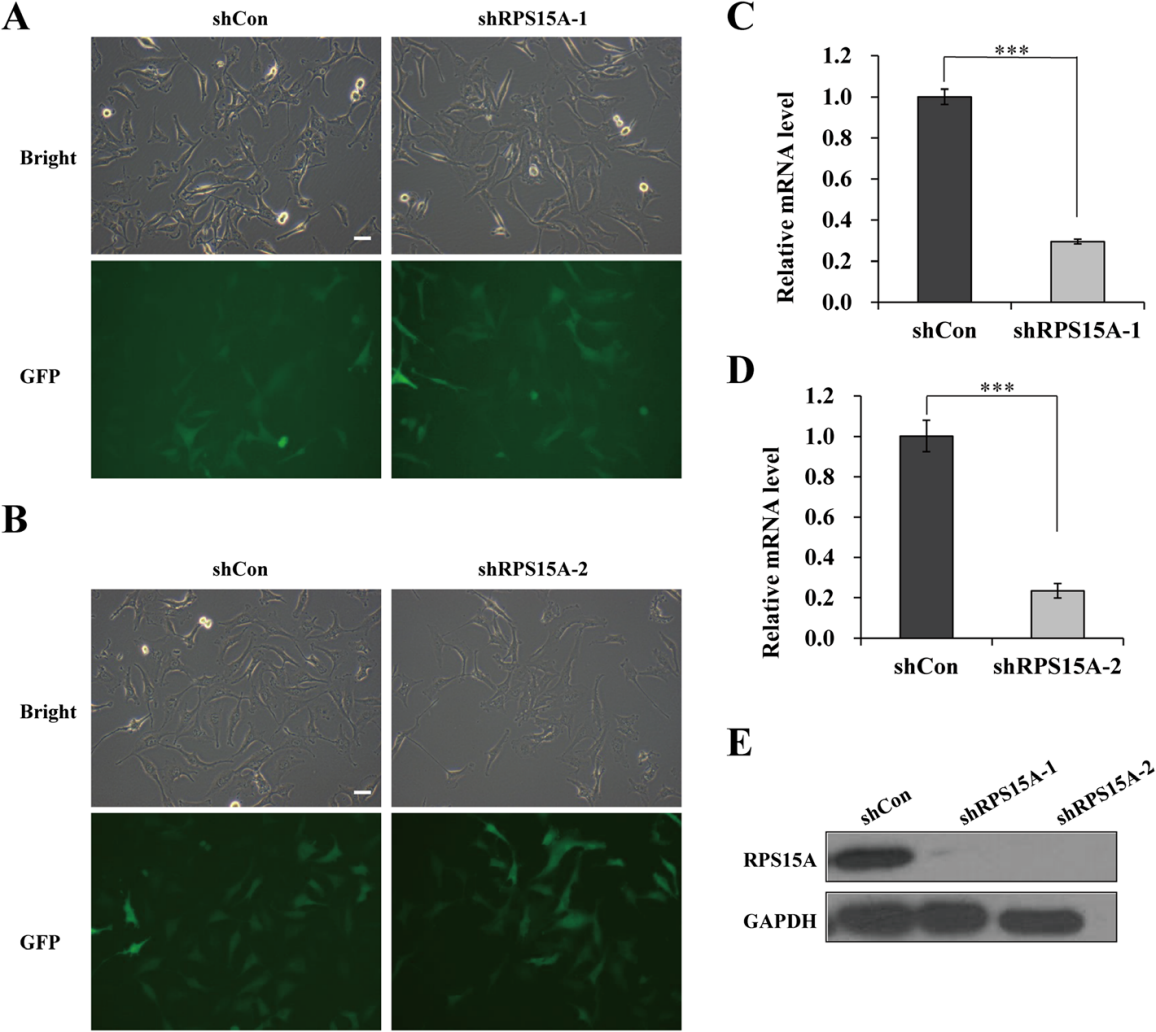
Verification of RPS15A silencing in U251 cells after shRPS15A infection. **a**, **b** Infection efficiency was assessed by counting GFP-positive cells with fluorescence microscopy. **c**, **d** The mRNA levels of RPS15A in U251 cells with three treatments (shCon, shRPS15A-1, and shRPS15A-2) measured by qPCR analysis. The experiment was performed in triplicate and repeated three times. **e** The protein levels of RPS15A in U251 cells with three treatments (shCon, shRPS15A-1, and shRPS15A-2) determined by Western blot analysis. ***p < 0.001

To investigate the biological function of RPS15A in glioblastoma cells, we constructed two individual shRNA vectors for RPS15A knockdown using lentiviral vectors carrying green fluorescent protein (GFP). Both control vectors (shCon) and two target vectors (shRPS15A-1 and shRPS15A-2) showed good infection rates 72 h post infection in U251 cells, determined by a fluorescence microscopy of the GFP expression (Fig. 1a, b). We performed RT-PCR and western blot to evaluate the efficiency of shRNA knockdown of RPS15A. As shown in Fig. 1c, d, both of two target shRNAs showed significant reduction of RPS15A mRNA compared to the shCon (*p* < 0.001). Western blot confirmed that, at protein level, target shRNAs efficiently knocked down RPS15A expression in U251 cells (Fig. 1e). Together, these results suggested that we successfully constructed RPS15A knockdown cell model, which could be applied to the following studies.

### RPS15A knockdown affects cell proliferation and colony formation ability

**Fig. 2:**
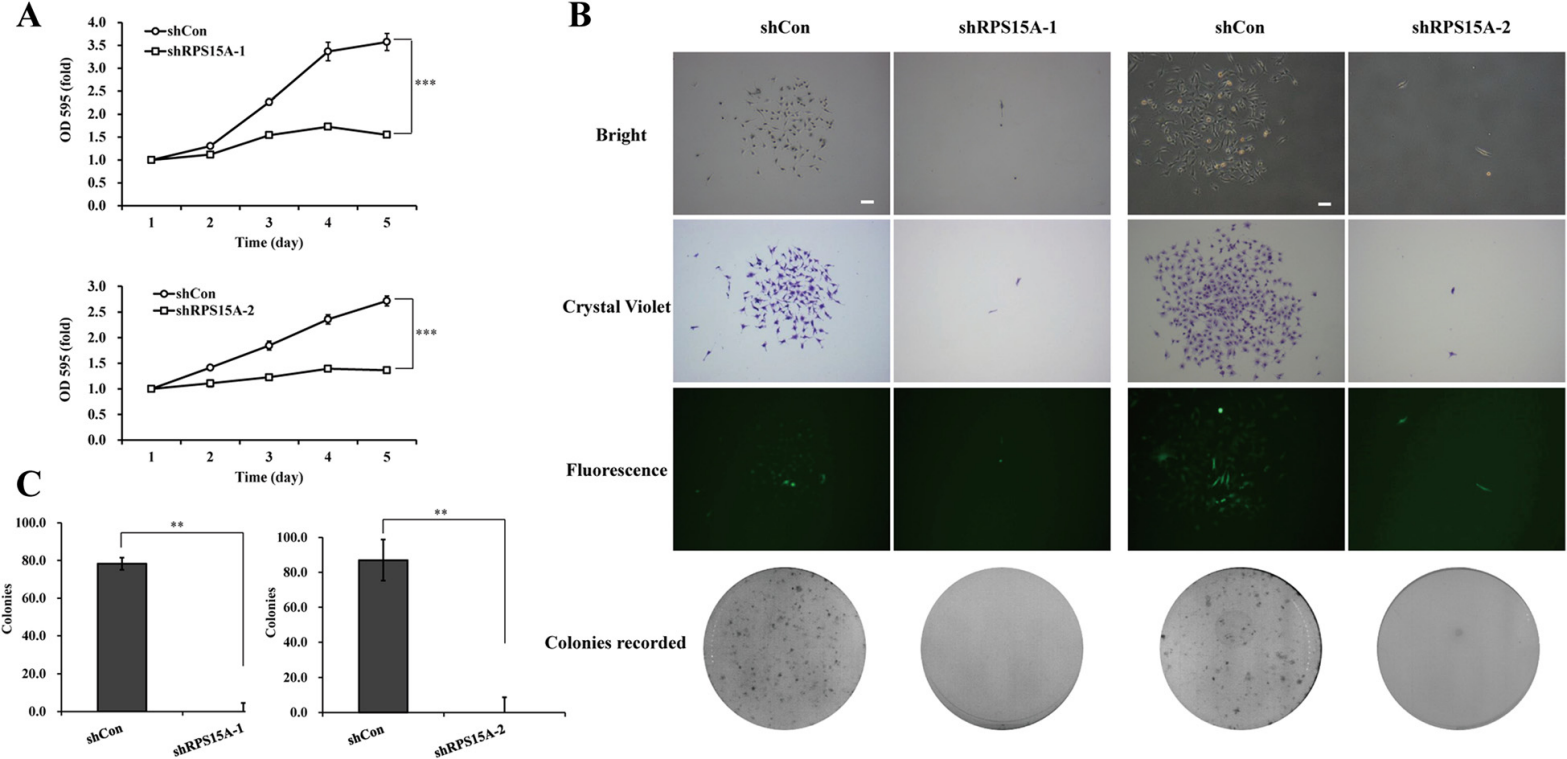
Lentivirus-mediated knockdown of RPS15A suppresses cell viability and the colony formation ability of U251 cells. **a** Growth curve of U251 cells with three treatments (shCon, shRPS15A-1, and shRPS15A-2) determined by MTT assay. **b** Representative images of colonies formed in U251 cells with three treatments (shCon, shRPS15A-1, and shRPS15A-2) measured by the colony formation assay. **c** Statistical analysis of colony formation ability as shown. The experiments was performed in triplicate and repeated three times. **p < 0.01, ***p < 0.001

We next examined the effect of RPS15A on cell proliferation and viability. We measured cell proliferation by MTT assay, a colorimetric assay assessing cell metabolic activity depending on NADPH oxidoreductase enzymes, over the course of 5 days. As shown in Fig. 2a, shRPS15A-treated cells showed significant inhibition of cell proliferation compared to cells treated with shCon (*p* < 0.001). We also did colony formation assay with cells treated by shCon vs. shRPS15A. As shown in Fig. 2b, c, shRPS15A-treated cells failed to form any colony, whereas shCon-treated cells can form colonies naturally (*p* < 0.01). Therefore, RPS15A could promote cell viability and proliferation ability.

### RPS15A plays a role in cell cycle progression in U251 cells

**Fig. 3:**
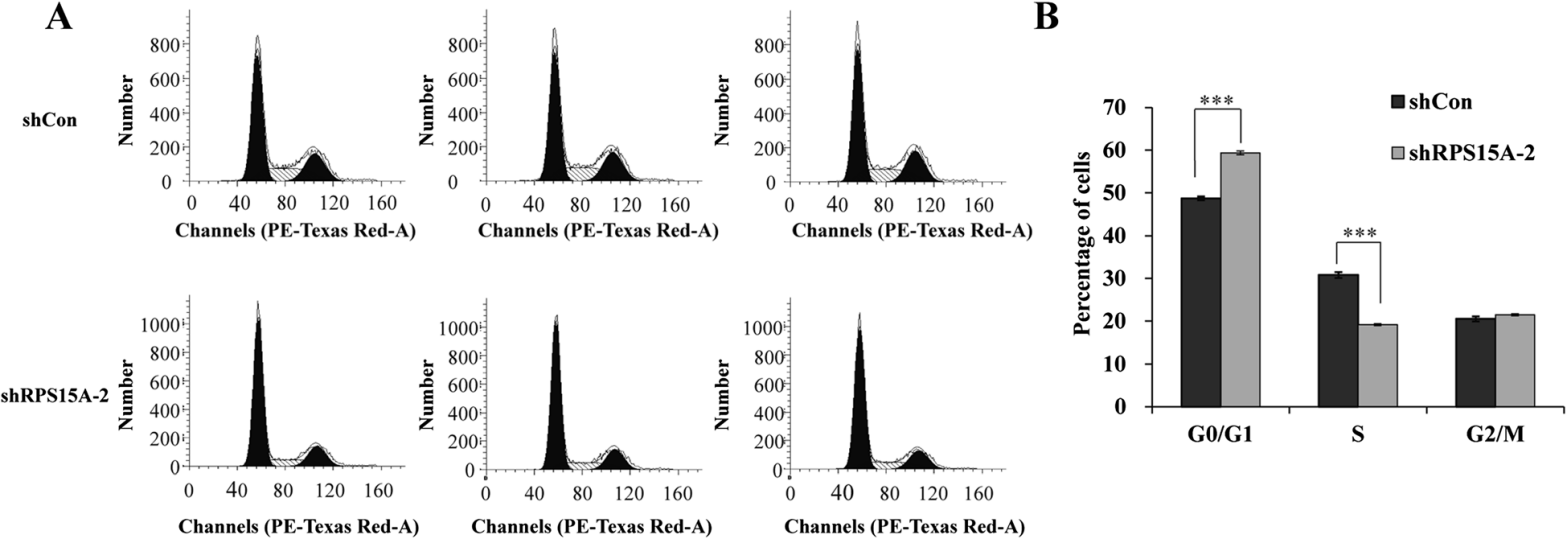
Lentivirus-mediated knockdown of RPS15A blocks the cell cycle progression of U251 cells. **a** Representative images of cell cycle distribution of U251 cells with two treatments (shCon and shRPS15A-2) analyzed by flow cytometry using PI staining. **b** Statistical analysis of the respective proportion of U251 cells in the G0/G1, S, and G2/M phases. The experiment was performed in triplicate and repeated three times. ***p < 0.001

Inhibition of cell proliferation could occur at any stage of the cell cycle. To investigate the mechanisms of the U251 cell proliferation inhibition induced by shRPS15A, we used flow cytometry to examine the cell cycle progression under the treatment of shCon or shRPS15A (Fig. 3a). We found that 72 h after shRNA treatment, shRPS15A-treated cells showed significantly reduced cells in S phase compared to shCon-treated cells (*p* < 0.001); instead, there were more cells accumulated in G0/G1 phase (*p* < 0.001) after shRPS15A infection, but no significant difference was found in G2/M phase (Fig. 3b). This cell cycle results strongly indicated that knockdown of RPS15A arrested cell cycle at G0/G1 phase, which means RPS15A plays an important role in cell cycle progress from G1 to S phase.

### RPS15A knockdown induces apoptosis in U251 cells

**Fig. 4:**
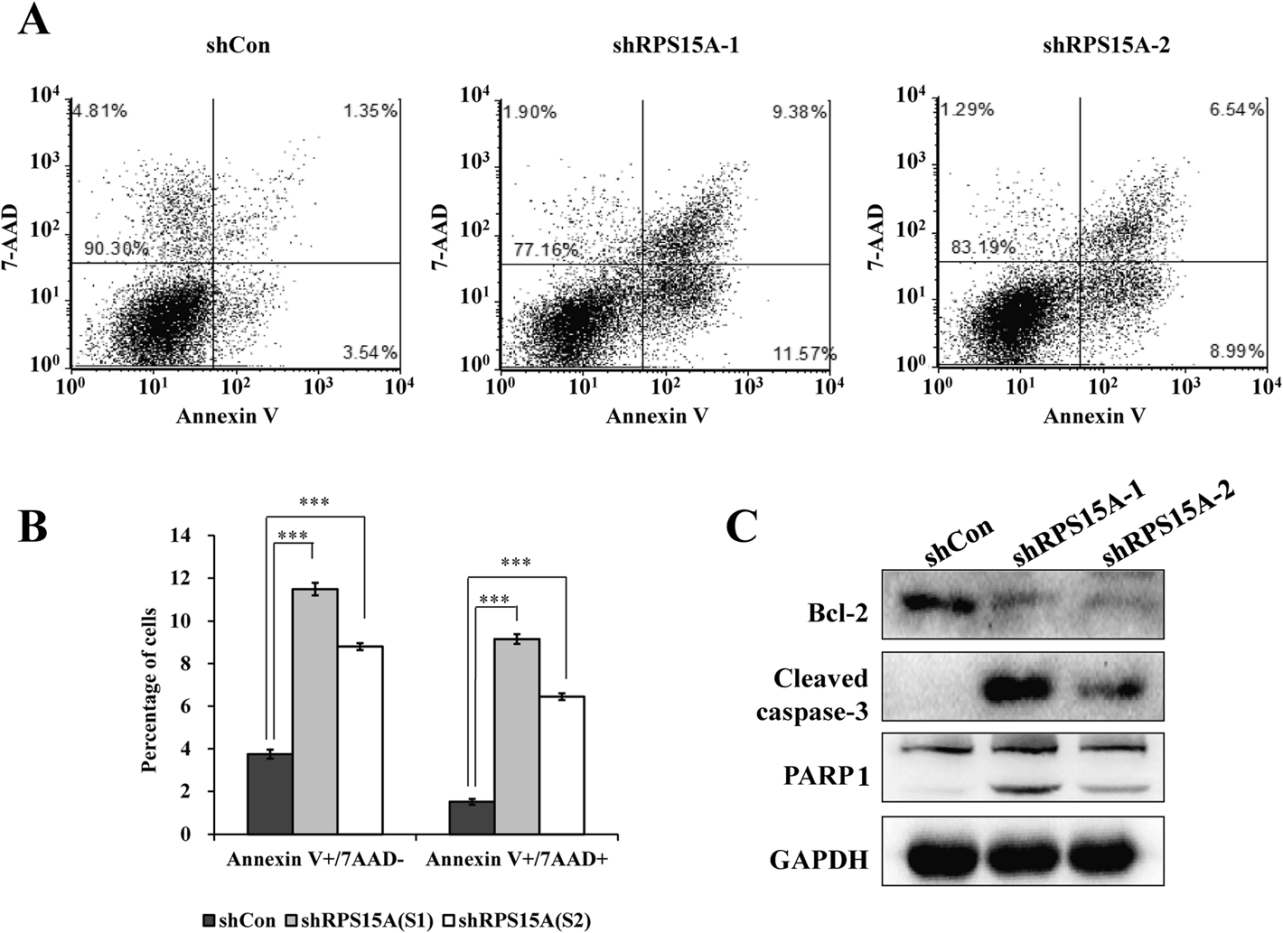
Knockdown of RPS15A induces cell apoptosis via activation of mitochondrial pathway. **a** Representative images of cell apoptosis of U251 cells with three treatments (shCon, shRPS15A-1, and shRPS15A-2) analyzed by flow cytometry using Annexin V/7-AAD double staining. **b** Statistical analysis of the proportions of U251 cells corresponding to early (Annexin V+/7-AAD−) and late (Annexin V+/7-AAD+) apoptotic cells. **c** Activation of bcl-2, caspase-3, and PARP signaling pathways measured by Western blot analysis. The experiment was performed in triplicate and repeated three times. ***p < 0.001

To further investigate the effect of RPS15A on cell apoptosis, Annexin V combined with 7-Aminoactinomycin D (7-AAD) staining was used to distinguish apoptosis (Fig. 4a) between early and late stages of apoptosis based on the exposure of phosphatidylserine to the surface of cell apoptosis [21]. We found that knockdown of RPS15A significantly induced cell early (Annexin V+/7-AAD−) and late apoptosis (Annexin V+/7-AAD+) in U251 cells (Fig. 4b, *p* < 0.001).

We further extended our study to investigate the pathway through which RPS15A knockdown induced apoptosis. Apoptosis is typically induced through two distinct signaling pathways: the death receptor pathway and mitochondrial pathway [22]. Because RPS15A is a cytosolic protein, we hypothesized it affected mitochondria pathway, in which bcl-2, caspase 3, and PARP are critical components. Thus, we carried out Western blot analysis to determine the expression of these three proteins in shRPS15A-treated cells. As shown in Fig. 4c, the anti-apoptotic and mitochondria localized protein Bcl-2 was down-regulated in U251 cells after RPS15A knockdown. The expression levels of cleaved caspase-3 and PARP were up-regulated in U251 cells following shRPS15A infection. This result indicated that RPS15A knockdown activated the mitochondrial pathway of apoptosis. What is more, shRPS15A-1 showed more strong effect on apoptosis induction than shRPS15A-2. Taken together, we found that RPS15A knockdown induced U251 cells apoptosis through mitochondrial pathway.

## Discussion

RPS15A encodes a 40S ribosomal protein S15a, one of the components of the 40S subunit in human [23]. Ribosomes are required for basic cellular functions, especially for cancer cells, whose growth and proliferation are uncontrolled, thus demand a large amount of protein synthesis [7]. GBM is invariably lethal glioma characterized by extremely high aggressiveness. However, little is known about the biological function of ribosome formation in GBM progression. In this study, we firstly evaluated the relative expression of RPS15A in glioma tissues and normal tissues and found it was significantly up-regulated in GBM tissues compared with that in normal tissues. To further investigate its role in GBM, the functional role of RPS15A in human GBM cell growth was studied by RNAi-mediated knockdown. Our results showed knockdown of RPS15A remarkably suppressed U251 cell growth, possibly due to induction of cell cycle arrest and apoptosis.

Similar effects of RPS15A were demonstrated in previous studies in other cancer types [13, 24]. Our study demonstrated that RPS15A was important for survival and proliferation of glioblastoma cancer cells. In the absence of this gene, the cells underwent apoptosis through intrinsic mitochondria pathway.

The mitochondrial pathway of apoptosis typically involves the release of mitochondrial cytochrome c into cytosol. The released cytochrome c combines with apoptotic protease activating factor 1 (Apaf-1) and procaspase-9, resulting in the activation of caspse-9 and caspase-3 [25]. Bcl-2 plays an important role in releasing cytochrome c from mitochondria. Our western blot result of Bcl-2 showed a significant decrease upon RPS15A knockdown. Thus, wastage of RPS15A led to the increase of anti-apoptotic signals (Bcl-2), which was in favor of the occurrence of apoptosis. Caspase-3 cleavage and PARP cleavage are the downstream of Bcl-2 [26, 27]. Our western blot confirmed these two cleavage events were both enhanced upon RPS15A knockdown. These results suggested that RPS15A knockdown induced apoptosis mainly through the activation of mitochondrial pathway. We hypothesize that RPS15A might be directly involved in the translation of Bcl-2 mRNA; thus, knockdown of RPS15A resulted in the down-regulation of Bcl-2 protein.

Many types of cancers, including glioblastoma, are resistant against many anti-cancer treatments. One of the main reasons is that they do not activate their intracellular apoptosis pathway [28]. Current standard chemotherapy treatment for glioblastoma is the use of alkylating agents, temozolomide, in combination with radiation therapy. Temozolomide has been proven somewhat effective in prolonging survival; however, tumor often returns after treatment, which begs the exploration of new drugs and therapies. Our finding of induction of apoptosis in GBM cell line by down-regulating RPS15A provides a new avenue, which targets ribosome proteins.

## Conclusions

From above analysis, we can see that RPS15A was significantly up-regulated in GBM tissues compared with that in normal tissues. Knockdown of RPS15A remarkably suppressed U251 cell growth; then, the results demonstrated that RPS15A was important for survival and proliferation of glioblastoma cancer cells. In the absence of this gene, the cells underwent apoptosis through activation of intrinsic mitochondria pathway.

In summary, our findings suggest RPS15A may play an important role in the progression of GBM and lentiviral-mediated silencing of RPS15A could be an effective tool in GBM treatment.

## References

[1] Kleihues P, Burger PC, Scheithauer BW (1993). The new WHO classification of brain tumours. Brain Pathol.

[2] Kleihues P, et al. The WHO classification of tumors of the nervous system. J Neuropathol Exp Neurol. 2002;61(3):215–25. discussion 226-9. 10.1093/jnen/61.3.215 11895036

[3] Kang TW, et al. Growth arrest and forced differentiation of human primary glioblastoma multiforme by a novel small molecule. Sci Rep. 2014;4:5546. 10.1038/srep05546 PMC4080225 24989033

[4] Jensen SA, et al. Spherical nucleic acid nanoparticle conjugates as an RNAi-based therapy for glioblastoma. Sci Transl Med. 2013;5(209):2124–34. 10.1126/scitranslmed.3006839 PMC4017940 24174328

[5] Stupp R, et al. Radiotherapy plus concomitant and adjuvant temozolomide for glioblastoma. N Engl J Med. 2005;352(10):987–96. 10.1056/NEJMoa043330 15758009

[6] Fatica A, Tollervey D (2002). Making ribosomes. Curr Opin Cell Biol.

[7] Ruggero D, Pandolfi PP (2003). Does the ribosome translate cancer?. Nat Rev Cancer.

[8] Ciarmatori S, et al. Overlapping functions of the pRb family in the regulation of rRNA synthesis. Mol Cell Biol. 2001;21(17):5806–14. 10.1128/MCB.21.17.5806-5814.2001 PMC87300 11486020

[9] Barna M, et al. Suppression of Myc oncogenic activity by ribosomal protein haploinsufficiency. Nature. 2008;456(7224):971–5. 10.1038/nature07449 PMC2880952 19011615

[10] Wool IG, Chan YL, Gluck A (1995). Structure and evolution of mammalian ribosomal proteins. Biochem Cell Biol.

[11] Chan YL, et al. The primary structure of rat ribosomal protein S15a. Biochem Biophys Res Commun. 1994;200(3):1498–504. 10.1006/bbrc.1994.1620 8185605

[12] Zhao X, et al. Decreased expression of RPS15A suppresses proliferation of lung cancer cells. Tumour Biol. 2015;36(9):6733-40. 10.1007/s13277-015-3371-9 25833696

[13] Zhang C, et al. Ribosomal protein S15A augments human osteosarcoma cell proliferation in vitro. Cancer Biother Radiopharm. 2014;29(10):451–6. 10.1089/cbr.2014.1698 PMC4267417 25409460

[14] Xu M, et al. Down-regulation of ribosomal protein S15A mRNA with a short hairpin RNA inhibits human hepatic cancer cell growth in vitro. Gene. 2014;536(1):84–9. 10.1016/j.gene.2013.11.075 24334120

[15] Rhodes DR, et al. ONCOMINE: a cancer microarray database and integrated data-mining platform. Neoplasia. 2004;6(1):1–6. 10.1016/s1476-5586(04)80047-2 PMC1635162 15068665

[16] Bredel M, et al. Functional network analysis reveals extended gliomagenesis pathway maps and three novel MYC-interacting genes in human gliomas. Cancer Res. 2005;65(19):8679–89. 10.1158/0008-5472.CAN-05-1204 16204036

[17] French PJ, et al. Gene expression profiles associated with treatment response in oligodendrogliomas. Cancer Res. 2005;65(24):11335–44. 10.1158/0008-5472.CAN-05-1886 16357140

[18] Murat A, et al. Stem cell-related “self-renewal” signature and high epidermal growth factor receptor expression associated with resistance to concomitant chemoradiotherapy in glioblastoma. J Clin Oncol. 2008;26(18):3015–24. 10.1200/JCO.2007.15.7164 18565887

[19] Shai R, et al. Gene expression profiling identifies molecular subtypes of gliomas. Oncogene. 2003;22(31):4918–23. 10.1038/sj.onc.1206753 12894235

[20] Sun L, et al. Neuronal and glioma-derived stem cell factor induces angiogenesis within the brain. Cancer Cell. 2006;9(4):287–300. 10.1016/j.ccr.2006.03.003 16616334

[21] Vermes I, et al. A novel assay for apoptosis. Flow cytometric detection of phosphatidylserine expression on early apoptotic cells using fluorescein labelled Annexin V. J Immunol Methods. 1995;184(1):39–51. 10.1016/0022-1759(95)00072-i 7622868

[22] Favaloro B, et al. Role of apoptosis in disease. Aging (Albany NY). 2012;4(5):330–49. 10.18632/aging.100459 PMC3384434 22683550

[23] Kenmochi N, et al. A map of 75 human ribosomal protein genes. Genome Res. 1998;8(5):509–23. 10.1101/gr.8.5.509 9582194

[24] Zhao X (2015). Decreased expression of RPS15A suppresses proliferation of lung cancer cells. Tumour Biol.

[25] Li P (1997). Cytochrome c and dATP-dependent formation of Apaf-1/caspase-9 complex initiates an apoptotic protease cascade. Cell.

[26] Swanton E (1999). Bcl-2 regulates a caspase-3/caspase-2 apoptotic cascade in cytosolic extracts. Oncogene.

[27] Perry DK (1997). Bcl-2 acts upstream of the PARP protease and prevents its activation. Cell Death Differ.

[28] Veenman L, Gavish M, Kugler W (2014). Apoptosis induction by erucylphosphohomocholine via the 18 kDa mitochondrial translocator protein: implications for cancer treatment. Anticancer Agents Med Chem.

